# Supplementary material for: Resolving the full spectrum of human genome variation using Linked-Reads
Source: Genome Res. 2019 Apr;29(4):635–45. doi: 10.1101/gr.234443.118 (PMC6442396; doi:10.1101/gr.234443.118)
Supplement: Supplemental Material [file supp_gr.234443.118_Supplemental_longranger-2.2.zip › longranger-2.2.tar.bz2!/lr2.2-public/lib/stan/cmdstan/stan/doc/api/html/num__elements_8hpp_source.html]

Stan: src/stan/math/matrix/num\_elements.hpp Source File


|  |  |
| --- | --- |
|  | Stan  2.5.0  probability, sampling & optimization |


- Main Page
- Related Pages
- Namespaces
- Classes
- Files

- File List
- File Members

All Classes Namespaces Files Functions Variables Typedefs Enumerations Enumerator Friends Macros Pages

- src
- stan
- math
- matrix

num\_elements.hpp

Go to the documentation of this file.

1 #ifndef STAN\_\_MATH\_\_MATRIX\_\_NUM\_ELEMENTS\_HPP

2 #define STAN\_\_MATH\_\_MATRIX\_\_NUM\_ELEMENTS\_HPP

3

4 #include <vector>

5 #include <stan/math/matrix/Eigen.hpp>

6

7 namespace stan {

8

9  namespace math {

10

17  template <typename T>

18  inline int

19  num\_elements(const T& x) {

20  return 1;

21  }

22

29  template <typename T, int R, int C>

30  inline int

31  num\_elements(const Eigen::Matrix<T,R,C>& m) {

32  return m.size();

33  }

34

43  template <typename T>

44  inline int

45  num\_elements(const std::vector<T>& v) {

46  if (v.size() == 0)

47  return 0;

48  return v.size() \* num\_elements(v[0]);

49  }

50

51  }

52 }

53 #endif

Eigen.hpp

stan::math::num\_elements

int num\_elements(const T &x)

Returns 1, the number of elements in a primitive type.

**Definition:** num\_elements.hpp:19


---

[ Stan Home Page ]

*© 2011–2014,
Stan Development Team.*
